# Generating adversarial examples without specifying a target model

**DOI:** 10.7717/peerj-cs.702

**Published:** 2021-09-13

**Authors:** Gaoming Yang, Mingwei Li, Xianjing Fang, Ji Zhang, Xingzhu Liang

**Affiliations:** 1 School of Computer Science and Engineering, Anhui University of Science and Technology, Huainan, China; 2 Department of Mathematics and Computing, University of Southern Queensland, Queensland, Australia

**Keywords:** Deep learning, Adversarial example, Generative adversarial networks, Adversarial machine learning

## Abstract

Adversarial examples are regarded as a security threat to deep learning models, and there are many ways to generate them. However, most existing methods require the query authority of the target during their work. In a more practical situation, the attacker will be easily detected because of too many queries, and this problem is especially obvious under the black-box setting. To solve the problem, we propose the Attack Without a Target Model (AWTM). Our algorithm does not specify any target model in generating adversarial examples, so it does not need to query the target. Experimental results show that it achieved a maximum attack success rate of 81.78% in the MNIST data set and 87.99% in the CIFAR-10 data set. In addition, it has a low time cost because it is a GAN-based method.

## Introduction

We have witnessed the unprecedented development of deep learning technologies in both academia and industry. It has been widely used in computer vision (Deng et al., 2019), natural language processing (Vijayakumar & Arun, 2019), and other fields (Ait-Khayi & Rus, 2019; Fischer & Krauss, 2018; Buhagiar, Zahir & Abhari, 2018; Cheng et al., 2016). However, recent research results show that deep neural networks are not safe when facing adversarial examples (Sharif et al., 2016; Zhong & Deng, 2020; Li et al., 2020b). In fact, attackers can make perturbed samples to mislead deep neural networks to produce the wrong output by adding appropriate noise to the normal samples.

Formally, adversarial attacks can be classified in different ways, such as an adversarial example may make the model produce false positives (Nguyen, Yosinski & Clune, 2015) or false negatives (Su, Vargas & Sakurai, 2019) from the perspective of the confusion matrix. In addition, depending on the attacker’s background knowledge, adversarial attacks are divided into white-box (Nguyen, Yosinski & Clune, 2015; Kurakin, Goodfellow & Bengio, 2016), gray-box (Zügner, Akbarnejad & Günnemann, 2018) and black-box (Papernot et al., 2017; Jia & Liang, 2017) settings. From the aspect of adversarial specificity, non-targeted (Su, Vargas & Sakurai, 2019) attacks only reduce the model’s credibility, while targeted attacks (Carlini & Wagner, 2017) mislead the model. Furthermore, the adversarial examples generated for misleading one model can also mislead other models, which is called attack transferability (Papernot, McDaniel & Goodfellow, 2016, Xie et al., 2019). Although adversarial examples are often discussed in classification tasks, attacks on generative models (Tabacof, Tavares & Valle, 2016) show that their existence is not related to the task domain of the model. Adversarial attacks are effective against a variety of data, including but not limited to images (Sharif et al., 2016), text (Liang et al., 2018), and audio (Carlini & Wagner, 2018).

Methodologically, there are also many types of adversarial attacks. Traditional adversarial attack include gradient-based methods and optimization-based methods. The main representative of the gradient-based method is fast gradient sign method (FGSM) (Goodfellow, Shlens & Szegedy, 2014). To calculate the gradient of the target model, the attacker needs to know their parameters. Moreover, the use of momentum (Dong et al., 2018) and gradient projection (Madry et al., 2017) in gradient-based methods will increase the attack success rate. The main representative of optimization-based methods is C&W (Carlini & Wagner, 2017), it regards they are generating adversarial examples as an optimization problem. In this type of method, the attacker obtains adversarial perturbation by setting multiple constraints. In addition, there are some unique methods. A few studies use deep neural networks to generate adversarial examples. For example, Mopuri et al. (2018) use a single generative neural network to obtain the adversarial perturbation against the entire dataset. Xiao et al. (2018) apply a generative adversarial network (Goodfellow et al., 2014) to generate high-quality adversarial examples. In recent work, the researchers used the quantified epistemic uncertainty of the deep learning model to replace the loss function in the traditional gradient-based method (Tuna, Catak & Eskil, 2021). Their experimental results show that attacks based on epistemic uncertainty are as powerful as conventional methods, which will bring new inspirations in the field of adversarial machine learning.

As we introduced above, the technology of adversarial attacks is evolving. Most existing research seems to focus on how to improve the success rate. However, a more practical problem is that existing methods need to query the target model in most cases, which makes them likely to be easily detected in real life. Therefore, ensuring the concealment of the attacker is an important topic. In fact, some research of black-box attacks reduces the number of queries (Li et al., 2020a) or avoids queries by transferability (Papernot, McDaniel & Goodfellow, 2016; Demontis et al., 2019). In this paper, we try to solve this problem from different angles. We propose the Attack Without a Target Model, a method to completely isolate the attack target from the generation process of adversarial examples, called Attack Without a Target Model (AWTM) for short. You only need to know the training set of the target. Our AWTM can attack the target without querying them at all. The contributions of this paper are summarized as follows:

- In order to ensure that the attacked model is invisible to the training of AWTM, we construct a random model to participate in the AWTM training process. In this way, our AWTM can generate adversarial examples without querying the target;
- In order to ensure that the samples generated by our AWTM can attack unknown target models, we studied the impact of classification boundaries on adversarial attacks. As a result, our AWTM generate more generalized adversarial examples, thus completing the attack without querying the target;
- In order to test the performance of AWTM, we attack multiple networks with different structures on both the MNIST and CIFAR-10 datasets. The success rate of AWTM reached 81.78% on MNIST and 87.99% on CIFAR-10. Experimental results show that our AWTM has a higher success rate than the transfer attack setting of some representative methods.

The structure of the remainder of this paper is as follows. In “Related Works”, we present the work related to adversarial attacks. In “Preliminaries”, we introduces the necessary background knowledge. And in “Proposing AWTM” and “Constructing AWTM”, we elaborated how AWTM was proposed and further instantiated. Then in “Training Logic”, we introduced the training of AWTM. The experimental results are reported in “Experiments and Results”.

## Related works

New methods always accompany theoretical research on adversarial examples, so this section briefly describes some representative approaches and the ideas they embody. Some of them is also prepared for later comparison experiments.

### Classic methods

The concept of adversarial examples was proposed by Szegedy et al. (2013). They use the L-BFGS method to find adversarial examples. This complex and slow-working method solves a box-constrained optimization problem to achieve targeted attacks in a white-box situation. Their research warns people: Neural networks can be easily fooled by slightly disturbed samples.

Goodfellow, Shlens & Szegedy (2014) proposed the FGSM, which is fast and straightforward. The attacker first calculates the gradient of the loss function concerning the input, and then they add some noise to the positive direction of the gradient. In this way, the processed samples can mislead the target model. Since FGSM is not iterative, its computational complexity is minimal. However, the perturbation it adds to the sample is significant most of the time. As one of the main contributions, FGSM represents an early view: the existence of adversarial examples is essentially due to the linearity of deep learning models.

Papernot et al. (2016) believe that the input sample contains some sensitive features. When passing through a neural network, the deviation of these features will be amplified layer by layer, resulting in completely different classification results. They proposed the Jacobian-based Saliency Map Attack (JSMA). The attacker uses the adversarial saliency map to find the sensitive features of the input and then adds perturbation to these features. The noise constructed by JSMA is quite accurate, and it can generate samples with high quality and high attack success rates.

Moosavi-Dezfooli, Fawzi & Frossard (2016) proposed the DeepFool, and it is an attack method based on classification boundaries. By linearly fitting the classification plane of the target model, the attacker iteratively adds noise to the sample until it is pushed across the plane. The idea of the DeepFool is very precious, but it costs a lot and works slowly.

Madry et al. (2017) proposed a projected gradient descent (PGD) attack and used it for adversarial training. Similar to the principle of FGSM, PGD is based on gradients. The difference is that it is an iterative method, and the gradient is projected rather than clipped in each step. Furthermore, the application of random restart also makes it easier to find adversarial examples. Although the PGD attack is powerful, like many iterative methods, its time cost is too high.

Carlini & Wagner (2017) proposed the C&W method. It treats the generation problem as an optimization for a normal sample under a two-distance constraint. The method notice that the adversarial example should be a class of samples: they are as close as possible to the normal sample point with the highest possible probability of classification error. The adversarial example perturbations generated by C&W attacks are quite accurate and extremely robust. It can maintain a high attack success rate against the model despite many existing defenses. However, this method costs a lot of time for each input, so it cannot be used for large-scale data.

Moosavi-Dezfooli et al. (2017) proposed the Universal Adversarial Perturbations. Unlike most adversarial attacks that target only a single sample, their method aims to find a universal perturbation for most examples in the data set. It can also be understood that the perturbation is about the target model rather than the sample.

### GAN-based methods

Xiao et al. (2018) proposed the advGAN, which is the most typical GAN-based attack method. In this framework, a generator *G* that receives normal samples *x* learns to construct a perturbation *G*(*x*), and *x* + *G*(*x*) is the adversarial example. While ensuring the attack success rate and the sample quality, the advGAN converts the repeated generation cost into a one-time training cost. Therefore, it can efficiently generate adversarial examples in batches.

Zhao, Dua & Singh (2017) turned their attention to the hidden space where the input is located. Their method is based on the WGAN framework (Arjovsky, Chintala & Bottou, 2017). Using latent space representation, the samples generated by this framework are more natural, and the perturbation has better interpretability.

Song et al. (2018) believe that the traditional generation method restricts the distance between the input sample and the adversarial example. They pointed out that these methods only search results in a small space. They constructed an auxiliary classifier that simulated the output of the human eye and used it as part of the ACGAN (Odena, Olah & Shlens, 2017) framework. Instead of limiting the results to the nearest neighbors of the input, their method directly searches for adversarial examples in the entire sample space.

## Preliminaries

This section introduced the necessary background about AWTM. Similar to advGAN, our method is also based on generative adversarial networks. And we add a pre-mapper to the AWTM framework, which is obtained through an autoencoder structure. The following briefly introduces the generative adversarial network and autoencoder.

### Generative adversarial networks

The Generative Adversarial Networks (GANs) is a powerful generative model, and two neural networks act as its generator and discriminator, respectively. The training step makes the two parts compete with each other, like an adversarial game under a maximum-minimum framework, and finally, the entire framework reaches a Nash equilibrium. The optimization function for generator *G* and discriminator *D* can be described as follows:

(1) $$minmaxV\left( {D,G} \right) = {E_{x{\rm \sim }{p_{data}}\left( x \right)}}\left[ {\log D\left( x \right)} \right] + {E_{z{\rm \sim }{p_z}\left( z \right)}}\left[ {\log \left( {1 - D\left( {G\left( z \right)} \right)} \right)} \right]$$

In general, the training of GANs often exhibits instability, and this problem can be solved by using WGAN (Arjovsky, Chintala & Bottou, 2017). While the loss function of the original GAN is derived from the JS divergence, WGAN uses the Wasserstein-1 distance to derive a new loss function. We prioritize WGAN when building the AWTM framework, which will help the generator face more complex tasks.

### Auto-Encoder

An auto-encoder is a special neural network structure that can be used for data dimensionality reduction or feature learning. It consists of two symmetrical neural networks, half of which are responsible for encoding and the other half for decoding (Hinton & Salakhutdinov, 2006). We apply the autoencoder as part of the model to obtain the mapper we need.

## Proposing awtm

This section gives the theoretical basis for constructing the AWTM framework. We start from the goal and get the theoretical prototype of AWTM step by step.

### Simulating changing classification boundaries

Our goal is to construct an attack method that does not specify a target model during the generation process. First, let us use a simple scene of the two-dimensional data to describe problems caused by the goal.

**Figure 1:**
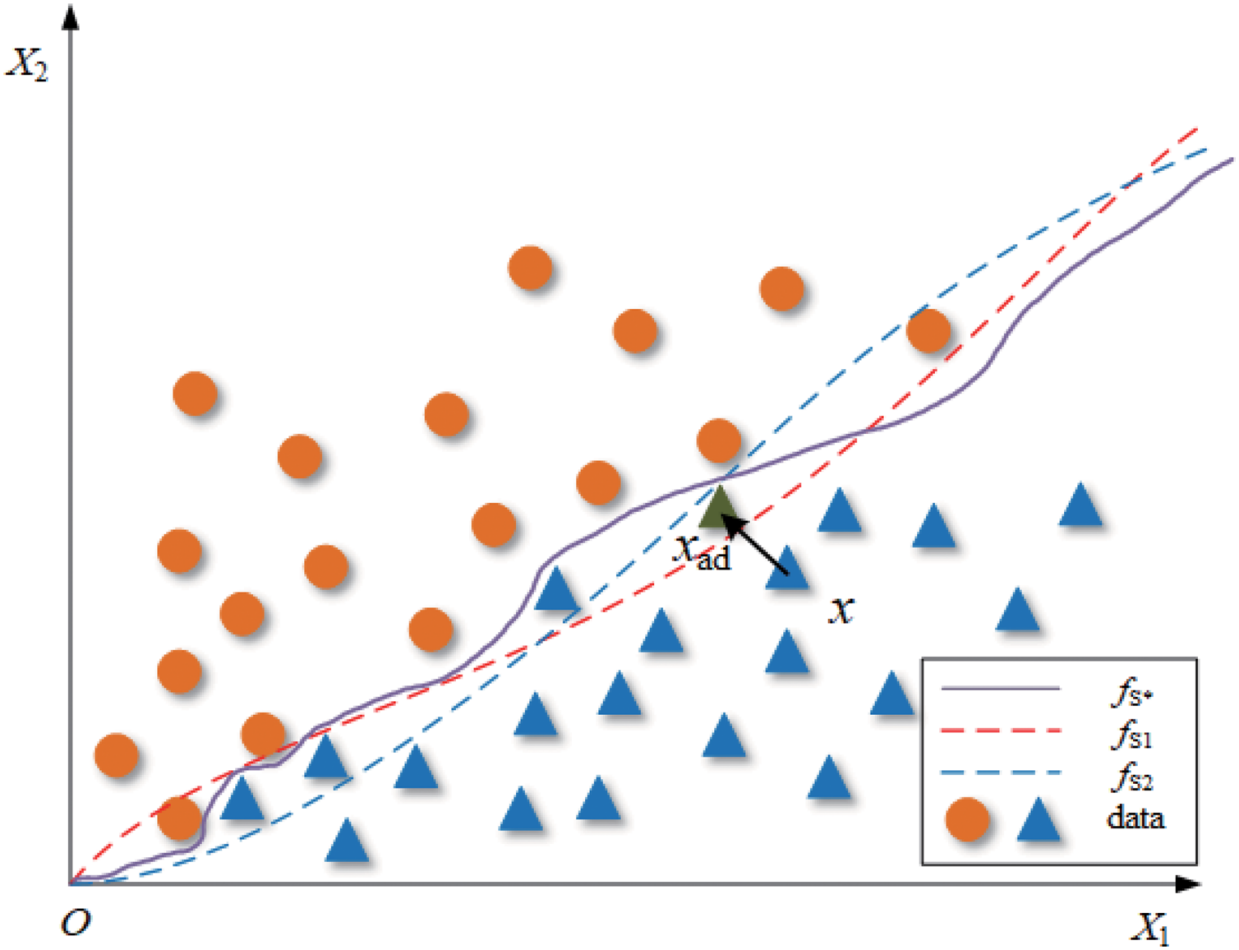
The classification boundary under a two-dimensional case.

Suppose there is an oracle classifier *f*_S*_ that can accurately divide the two-dimensional data set *S* into two classes, *f*_*S*1_ and *f*_*S*2_ are ordinary classifiers trained on *S*, and their classification boundaries are shown in Fig. 1. For data set *S*, the data distribution of the data set is determined, but the classification boundaries of the classifiers trained on it are not the same. Therefore, we can infer that the boundary of *f*_*S*1_ and *f*_*S*2_ is close to but not the same as *f*_S*_. For a common adversarial attack algorithm *A*, the process of generating adversarial examples for *f*_*S*1_ can be written as follows:

(2) $$A\left( {x,{f_{S1}}} \right) = {x_{ad}}$$

where *x* is the clean sample from *S*, and *x*_*ad*_ is the adversarial example. This process is shown in Fig. 1 as “*x* crosses the classification boundary of *f*_*S*1_”.

**Figure 2:**
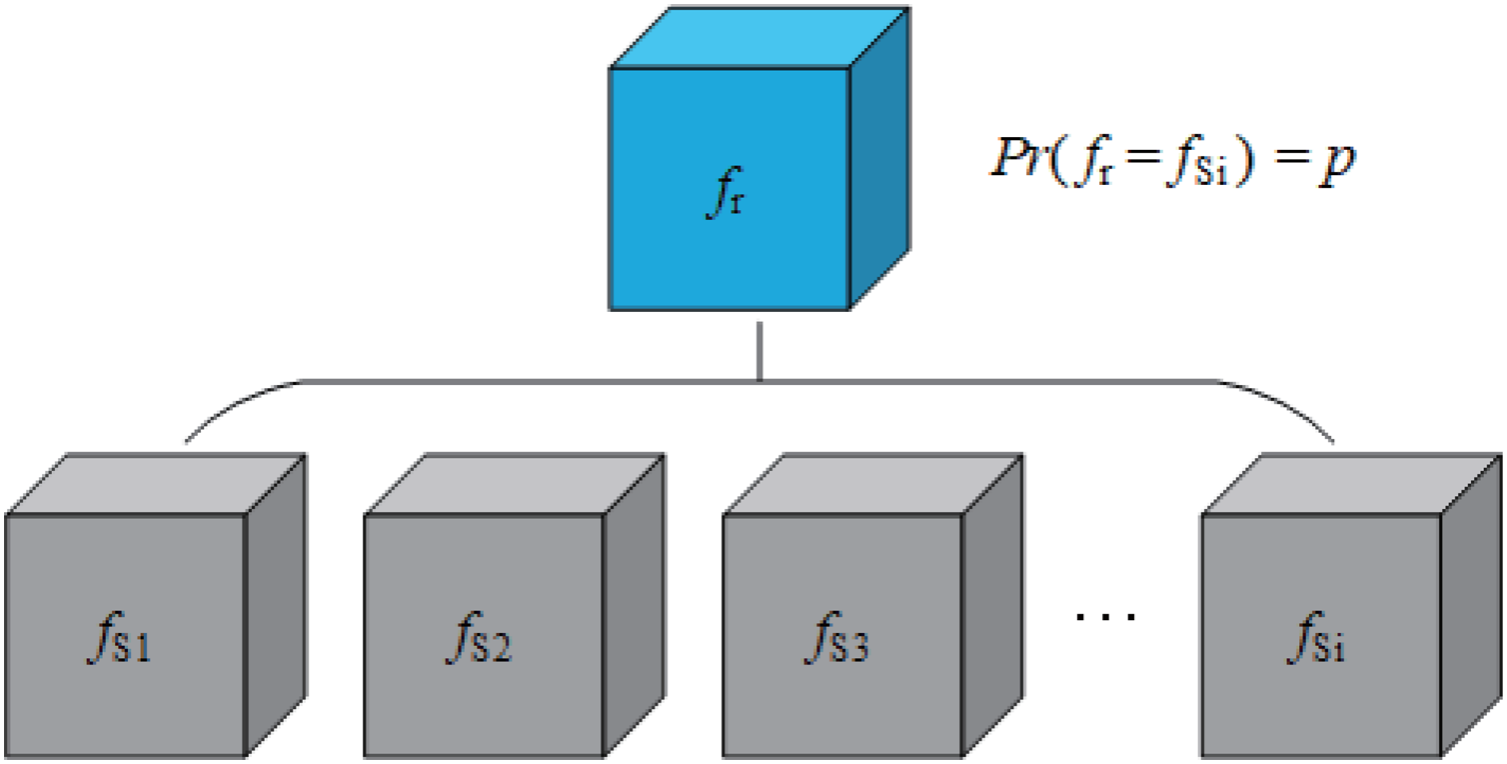
A solution to simulate changing classification boundaries.

Now we consider how to construct AWTM. Removing *f*_*S*1_ in Eq. (2) and no longer providing a definite classification boundary for the attack algorithm *A* is a straightforward solution to our goal. Unfortunately, this solution makes it difficult for us to define the loss function accurately. While ensuring that the target of the attack is entirely invisible, we have to increase the possibility of a successful attack as much as possible. Then another solution is finding an alternative target *f*_*Sn*_, which is not the real target but can provide a more representative classification boundary, so that the samples generated for this boundary can also cross other boundaries with the greatest probability. However, the classification boundaries corresponding to multiple classifiers trained on a particular data set are not sure. They may be affected by various factors such as model structure and training process. It is difficult to find out which one of them is “the most representative”. Therefore, it is necessary to consider the uncertainty of the classification boundary when generating adversarial examples. In this way, we can build a classifier *f*_*r*_ that can change its classification boundary with a probability. Let *f*_*r*_ replace the classifier with a definite boundary as the target of attack, so that different classification boundaries can be considered in the generation process. Fig. 2 shows the theoretical structure of *f*_*r*_, and our strategy looks like this:

(3) $$A\left( {x,{f_r}} \right) = {x_{ad}},Pr\left( {{f_r} = {f_{Si}}} \right) = p$$

Here *f*_*r*_ is also the theoretical prototype of the random classifier mentioned in “Constructing AWTM”. However, it brings a new problem: From the perspective of batch generation, the attack algorithm *A* does consider various boundaries, but for a single sample, the attack algorithm *A* still only considers one of the boundaries with the probability *p*.

### Constructing attack algorithm with a deep generative model

So, how to consider multiple classification boundaries in the process of generating each sample? We need algorithm *A* to add the information of boundary changes to each generation step. It sounds complicated. However, deep generative models provide the possibility to achieve this goal. Let us assume that *A*_*θ*_ is the generator of the deep generative model, and *θ* is the internal parameter of *A*. Each batch of adversarial examples generated by *A*_*θ*_ will be evaluated by the metric *L*, and *L* contains the information of the changing boundary. Therefore, as long as we use *L* feedback to adjust *θ*, we can make *A*_*θ*_ close to our needs:

(4) $${\theta} = argmax\;L\left( {{x_{ad}},x} \right)\;where\;{x_{ad}} = {A_\theta }\left( {x,{f_r}} \right)$$

In fact, AWTM is based on the GAN framework, which is an excellent deep generative model.

## Constructing awtm

**Figure 3:**
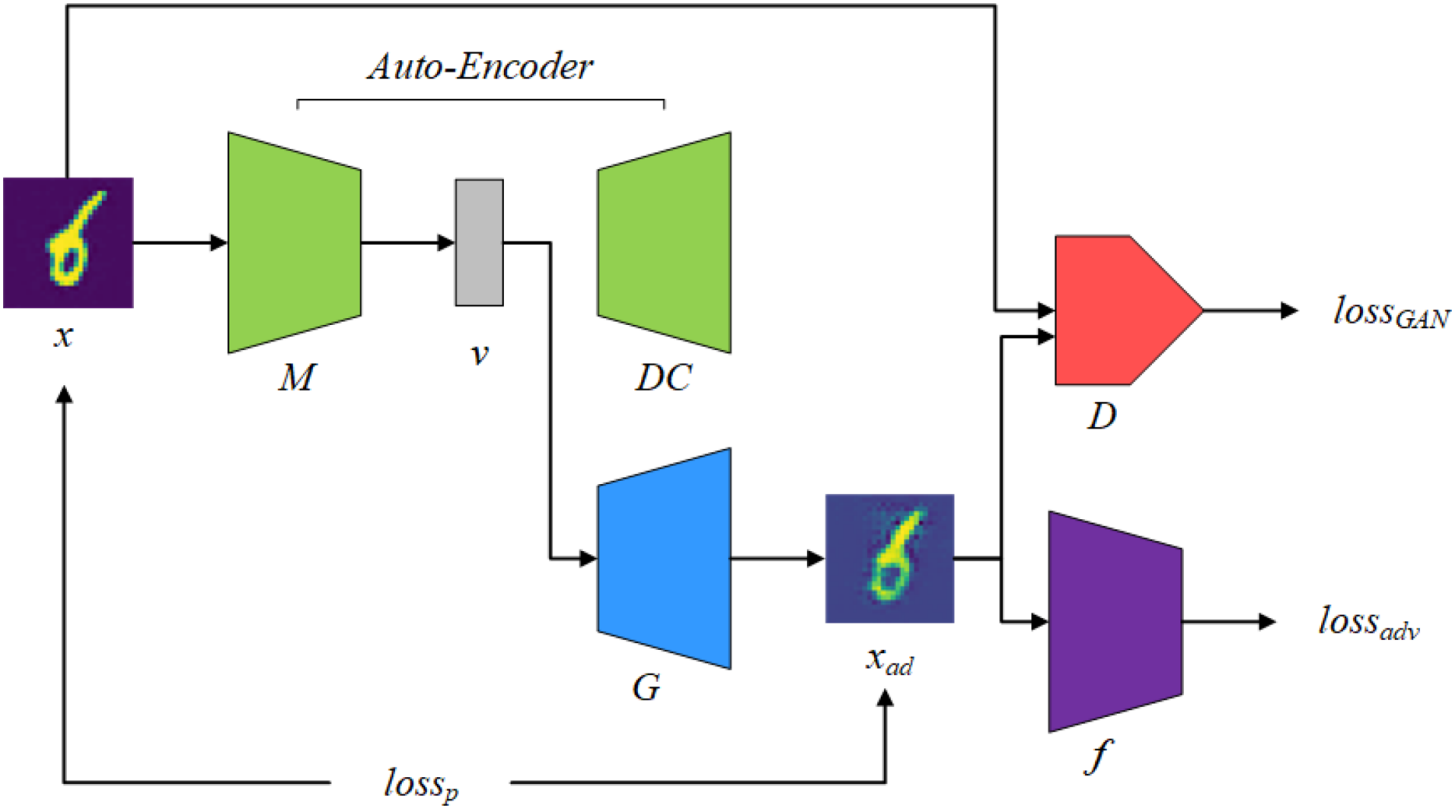
The AWTM threat model.

This section will introduce how to instantiate the previous ideas into the various components of AWTM and how they work. The AWTM framework is shown in Fig. 3. The mapper *M* maps a normal sample *x* to a dense vector *v* under a high-dimensional feature space ${\mathbb R}^n$ and the generator *G* processes *v* into an adversarial example *x*_*ad*_. The AWTM is a grey-box, non-targeted attack method, and it only needs to know the training dataset of the target. It does not require the attacker to have any information about the model parameters.

### The random classifier *f*

**Figure 4:**
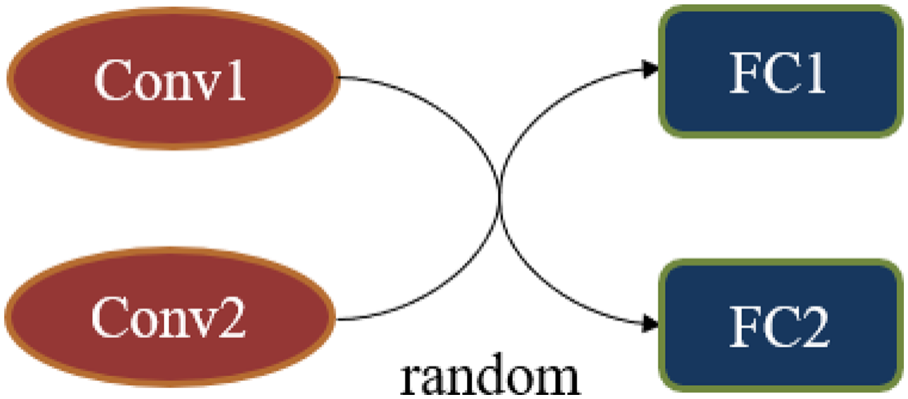
The construction of the random classifier *f*.

In the subsection “Simulating Changing Classification Boundaries”, we considered a classifier that can change the classification boundaries. A simple method to achieve it is to sample with a certain probability in a set of classifiers. However, this method is very inefficient, and we have adopted a more flexible method when implementing *f*. The essence of *f* is a classification network whose structure can produce some random variation. Figure 4 is an example of a random classifier, there is a random combination between two convolutional blocks and fully connected layers, all of the convolutional blocks are structurally different.

### The mapper *M*

As an auxiliary component, the mapper *M* is optional, and it is not used in advGAN. In our AWTM, the generator *G* accomplishes a very complex task. It is difficult to directly use the sample as the input of *G* and map it to space where the adversarial example is located, so we use the mapper *M* to make it the generating task easier. The *M* filters out the redundant information in the sample and only retains the key features related to the semantics of the input. By using the dense vector *v* as the input of the generator *G*, the model capacity requirement for *G* is reduced. Similar to advGAN, the time cost of AWTM is mainly focused on the training phase, and the use of *M* also helps to reduce the training cost.

We obtain the mapper *M* required by the framework through an auto-encoder structure. In Fig. 3, the mapper *M* and the decoder *DC* form an autoencoder structure, which will participate in the training process of the generator *G*. Note that the structural design of *M* should be flexible, because the dimension of the feature vector *v* will change according to the complexity of the input data.

### Generative adversarial networks in our method

We have explained the necessity of using a deep generative model in the section “Construct Attack Algorithm with Deep Generative Model”. The GAN model should be our first choice, because it has made considerable progress in recent years. In addition, as a successful precedent for applying GAN to adversarial sample generation, advGAN can also provide experience for completing our AWTM. In fact, we refer to the structure of the advGAN when designing AWTM except that the generator *G* of AWTM takes a dense vector instead of random noise as the input.

The tasks of the generator *G* include two aspects: First, for a set of features *v* extracted by the mapper *M*, *G* reconstructs them in a low-dimensional space. This task is completed under the supervision of the discriminator *D*. Second, in the reconstruction process, *G* needs to consider making the result cross the boundary of the random classifier *f*. This task is assisted by the adversarial loss provided by *f*. Note that these two tasks correspond to *loss*_*GAN*_ and *loss*_*adv*_ in Fig. 3, respectively. In addition, *loss*_*p*_ is used to limit the distance between the generated result and the original sample. These three loss functions will be explained in detail in the “Training Logic” section.

## Training logic

The training process of AWTM follows the WGAN framework. After the mapping of *M*, the input is compressed into a dense vector *v* in a high-dimensional space ${{\rm {\mathbb R}}^n}$:

(5) $${v} = M\left( x \right),\quad{v} \in {{\rm {\mathbb R}}^n}$$

The generator *G* remaps ***v*** to a low-dimensional space. The goal of discriminator *D* is to distinguish as much as possible between the samples from the generator *G* and the real dataset, giving low scores to the former and high scores to the latter, while the goal of *G* is to maximize the score given by *D* for the generated samples. In this competitive mode, *G* will learn to generate more realistic data distribution. Here WGAN’s loss function:

(6) $$los{s_{GAN}} = {E_{x{\rm \sim }p\left( x \right)}}\left[ {D\left( x \right)} \right] - {E_{{v}{\rm \sim }p\left( {v} \right)}}\left[ {D\left( {G\left( {v} \right)} \right)} \right]$$

The random classification model *f* leads generator *G* to construct adversarial features by using a loss function between its output *x*_*ad*_ and the true label *y*:

(7) $$los{s_{adv}} = {J_f}\left( {{x_{ad}},y} \right) = {J_f}\left( {G\left( {v} \right),y} \right)$$

And the distance metric *J*_*f*_ is referred to Carlini & Wagner (2017):

(8) $${J_f}\left( {G\left( {v} \right),y} \right) = \max \left( {\max \{ {Z_f}{{(G({v}))}_i}:i \ne y\} - {Z_f}{{(G({v}))}_y},0} \right)$$

where *Z*_*f*_ is the last hidden layer of *f*. Due to the special structure of *f*, its performance in each iteration is random, which makes *G* learning adversarial features more generally.

To make the generated sample as close as possible to the original sample, we create a loss function between the output of *G* and the original sample, using a *L*_2_-norm to constrain them in the input space to a range close to the original sample. Here, the loss is defined as:

(9) $$los{s_p} = {\rm \parallel }{x_{ad}} - x{{\rm \parallel }_2} = {\rm \parallel }G\left( {v} \right) - x{{\rm \parallel }_2}$$

Finally, the loss function for the entire model is the band-weighted sum of the above losses:

(10) $$L = los{s_{GAN}} + \alpha los{s_{adv}} + \beta los{s_p}$$

There are two hyperparameters *α* and *β*. The *α* is similar to the attack coefficient in the traditional method, which indicates the strength of the attack. In general, the *α* is negatively correlated with the quality of the sample; The *β* controls the difference between the generated sample and the original sample under the input space. A larger *β* causes the generated sample to be more similar to the original one. The AWTM training process is based on Algorithm 1.

**Algorithm 1:** AWTM training.

**Input:** the training set *T*
**Output** the mapper *M*, the generator *G*
1 Initialize the mapper *M*, the decoder *DC*, the discriminator *D*, and the generator *G;*
2 **for** *iterations* ≤ *epochs* **do**
3 Sampling a batch of samples $\left( {{x^{(1)}},{x^{(2)}}, \ldots {x^{(i)}}} \right)$ from the training set *T*, and
$\left( {{y^{(1)}},{y^{(2)}}, \ldots {y^{(i)}}} \right)$ is the corresponding label;
4 Train the autoencoder structure formed by the mapper *M* and the *DC* decoder to
minimize $\displaystyle{1 \over n}\sum\nolimits_{i = 1}^n {(M\left( {{x^{(i)}}} \right) - {x^{(i)}})^2}$
5 Fix the parameters of *M*, the generator *G* and the mapper *M* use $\left( {{x^{(1)}},{x^{(2)}}, \ldots {x^{(i)}}} \right)$ to
generate a batch of adversarial examples $x_{ad}^{(i)} = G(M({x^{(i)}}))$;
6 Fix the parameters of *G*, use the normal sample *X* and the generated sample
$\left( {x_{ad}^{(1)},x_{ad}^{(2)}, \ldots x_{ad}^{(i)}} \right)$ to train the discriminator *D*, s.t.
7 Fixing the parameters of *D*, update the parameters of *G*, s.t.
$\min \displaystyle{1 \over n}\mathop {\sum\nolimits_{i = 1}^n }\nolimits_ \left( { - D\left( {x_{ad}^{(i)}} \right) + {\rm \parallel }x_{ad}^{(i)} - {x^{(i)}}{{\rm \parallel }_2} + {J_f}\left( {M({x^{(i)}}),{y^{(i)}}} \right)} \right)$
8 **end**

## Experiments and results

This section explains the experimental details. We trained AWTM on different datasets, and conducted multiple sets of experiments to compare the generation results, time cost, and attack success rate of AWTM and several existing methods.

### Experimental setup

#### Datasets

We trained the AWTM threat model on the MNIST and CIFAR-10 datasets, respectively. The MNIST dataset consists of 70,000 labeled 0–9 handwritten numeric samples, each of which is a single-channel image of 28 × 28 pixels in size. The test set contains 60,000 images, and the other 10,000 images constitute the test set. The MNIST dataset refers to http://yann.lecun.com/exdb/mnist/. In contrast, the CIFAR-10 dataset is more complex and consists of 60,000 images of objects with labels, each with three channels and a size of 32 × 32 pixels. The test set contains 50,000 images, while the other 10,000 are used as the test images. The CIFAR-10 dataset refers to https://www.cs.toronto.edu/~kriz/cifar.html.

#### Target models

In this work, since the AWTM does not specify a particular model as a target, we hope that the samples it generates are effective for most architectures. Therefore, we trained three different networks as the test models. The AWTM generates samples only once and then tests the attack success rate achieved by those samples on the three target models separately. On the MNIST training set, the three target models FNN (1024-4096-1024), LeNet, and ResNet-18 are trained with *epoch* = 100. We obtained the following classification accuracy on the test set: the FNN (82.80%), LeNet (96.28%), ResNet-18 (99.22%). Here, the FNN has a lower capacity, so its accuracy is also lower. On the CIFAR-10 training set, the target models and their accuracy are CNN (82.54%), ResNet-9 (85.09%), ResNet-34 (89.22%).

#### AWTM main framework

When we construct the threat model, the generator *G* used in the experiment is almost the same as the decoder *DC* architecture. The mapper *M* and the decoder *DC* form the autoencoder structure, and the mapping rules they learn should be paired. We not only expect that *G* learn to decode the dense vector ***v***, but also need *G* to be able to construct adversarial features in it. Therefore, we want the *G* to have a capacity greater or equal to the *M*. Note that depending on the complexity of the data set, the dense vector ***v*** we choose on the two data sets has different dimensions. The threat models we constructed on the two datasets are slightly different. Tables 1 and 2 show the structure of the threat model and the dimension of *v* we used in the experiment.

**Table 1:** The architecture of the threat model on MNIST.

GAN framework		Autoencoder (${v} \in {{\rm {\mathbb R}}^{100}}$)	
Generator G	Discriminator D	Encoder M	Dncoder DC
Deconv(256, 4, 1, 0) LeakyReLU	Conv(64, 5, 2, 2) ReLU	Conv(64, 5, 2, 2) ReLU	Deconv(256, 4, 1, 0) LeakyReLU
Deconv(128, 3, 2, 1) LeakyReLU	Conv(128, 3, 2, 1) ReLU	Conv(128, 3, 2, 1) ReLU	Deconv(128, 3, 2, 1) LeakyReLU
Deconv(64, 3, 2, 1) LeakyReLU	Conv(256, 3, 2, 1) ReLU	Conv(256, 3, 2, 1) ReLU	Deconv(64, 3, 2, 1) LeakyReLU
Deconv(1, 4, 1, 0) Tanh	Conv(1, 4, 1, 0)	FC(4096, 1024) ReLU FC(1024, 100)	Deconv(1, 4, 1, 0) Tanh

**Table 2:** The architecture of the threat model on CIFAR-10.

GAN framework		Autoencoder (${v} \in {{\rm {\mathbb R}}^{4,096}}$)	
Generator G	Discriminator D	Encoder M	Dncoder DC
Deconv(128, 4, 2, 1) LeakyReLU	Conv(64, 4, 2, 1) BN + ReLU	Conv(64, 4, 2, 1) ReLU	Deconv(128, 4, 2, 1) ReLU
Deconv(64, 4, 2, 1) LeakyReLU	Conv(128, 4, 2, 1) BN + ReLU	Conv(128, 4, 2, 1) ReLU	Deconv(64, 4, 2, 1) ReLU
Deconv(3, 4, 2, 1) Tanh	Conv(256, 4, 2, 1) BN + ReLU Conv(1, 4, 1, 0)	Conv(256, 4, 2, 1) ReLU	Deconv(3, 4, 2, 1) ReLU

#### The random classifier *f*

The structure of the random classifier is shown in Tables 3 and 4, and its random part is selected each time with a 50% probability as the network used for the current batch during forwarding propagation.

**Table 3:** The architecture of the random classifier on MNIST.

Convolution block		Fully connected block	
Conv1	Conv2	FC1	FC2
Conv(64, 5, 2, 2) BN + ReLU	Conv(64, 3, 2, 0) BN + ReLU	FC(4096, 1024) ReLU	FC(4096, 1024) ReLU
Conv(128, 3, 2, 1) BN + ReLU	Conv(128, 3, 2, 1) BN + ReLU	FC(1024, 1024) Dropout(0.5)	FC(1024, 1024) Dropout(0.5)
Conv(256, 3, 2, 1) ReLU	Conv(256, 3, 2, 1) + ReLU	ReLU FC(1024, 10) Sigmoid	ReLU FC(1024, 10) Sigmoid

**Table 4:** The architecture of the random classifier on CIFAR-10.

Convolution block		Fully connected block	
Conv1	Conv2	FC1	FC2
Conv(32, 3, 1, 1) BN+ReLU Conv(32, 3, 1, 1)	Conv(32, 5, 1, 2) BN + ReLU Conv(32, 5, 1, 2)	FC(2048, 512) BN + ReLU FC(512, 10)	FC(2048, 512) BN + ReLU FC(512, 10)
BN + ReLU + MaxPool(2, 2) Conv(64, 3, 1, 1) BN + ReLU Conv(64, 3, 1, 1)	BN + ReLU + MaxPool(2, 2) Conv(64, 5, 1, 2) BN + ReLU Conv(64, 5, 1, 2)		
BN + ReLU + MaxPool(2, 2) Conv(128, 3, 1, 1) BN + ReLU Conv(128, 3, 1, 1) BN + ReLU + MaxPool(2, 2)	BN + ReLU + MaxPool(2, 2) Conv(128, 5, 1, 2) BN + ReLU Conv(128, 5, 1, 2) BN + ReLU+MaxPool(2, 2)		

In principle, before training the threat model, please ensure that the random classifier *f* has been trained. Although the structure of the random classifier is different from the common deep learning classifier, there is no difference in the training algorithm. The only thing to note is that the structure of the random classifier should keep changing during the training process. In addition, using pre-training parameters on the convolution block will help the training process.

### Training process

**Figure 5:**
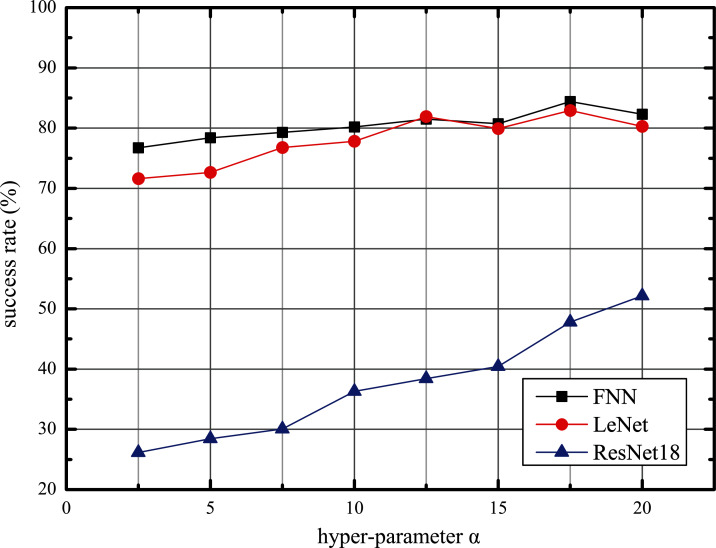
The adversarial tendency of the samples generated by AWTM as *α* increases.

Using the above structure, we follow Algorithm 5 to train our AWTM. Furthermore, we observed the impact of hyperparameter *α* on the performance of AWTM attacks. Taking the MNIST data set as an example, we fix *β* = 1 and adjust the value of *α* to obtain different threat models and calculate the success rate against the three target models on the test set. As shown in Fig. 5, the scale of *α* is positively correlated with the attack success rate. However, an excessively large *α* will reduce the quality of the generated samples and affect the stability of the training process.

### Generate adversarial examples

**Figure 6:**
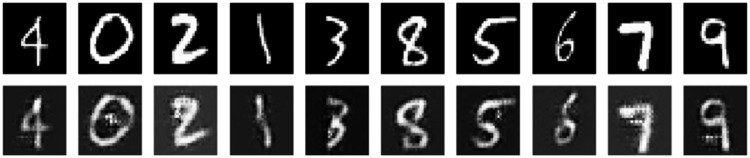
Normal samples (top) and adversarial examples (bottom) on MNIST.

**Figure 7:**
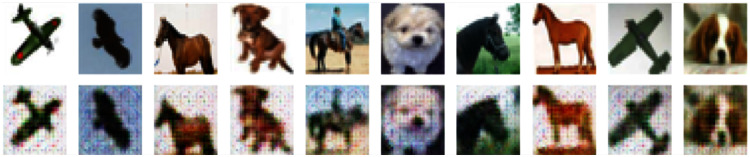
Normal samples (top) and adversarial examples (bottom) on CIFAR-10.

After training for 100 epochs, we get the mapper *M* and the generator *G*. Now we can use them to generate adversarial examples. On the MNIST and CIFAR-10 datasets, the results are shown in Figs. 6 and 7.

To compare the attack effects of these samples, we tested the attack success rate on two datasets and benchmarked the performance with several existing methods. Their implementations are all from advbox (Goodman et al., 2020), and their parameters are given in the Appendix A. In addition, All the attack methods involved in the experiment used the entire MNIST and CIFAR-10 test set.

**Table 12:** All algorithm’s parameters in our experiments.

Method	Parameter	Value (MNIST)	Value (CIFAR-10)
FGSM	Epsilons	0.1	0.05
JSMA	Theta	0.5	0.3
	Max perturbations per pixel	9	7
	Max iterations	1,000	1,000
DEEPFOOL	Overshoot	0.8	7
	Iterations	5	100
PGD	Epsilons	0.005	0.001
	Iterations	3	3
CW	Initial const	100	100
	Binary search step	4	1
	Max iterations	1,000	500
AdvGAN	Learning rate of G	0.0005	0.0005
	Learning rate of D	0.0001	0.0001
	Epochs	100	100
	Batch size	128	128
	*α*	10	0.1
	*β*	1	1
AWTM	Learning rate of G	0.0005	0.0005
	Learning rate of D	0.0001	0.0001
	Learning rate of M	0.0001	0.0001
	Epochs	100	100
	Batch size	128	128
	*α*	10	0.1
	*β*	1	1

We divide an adversarial attack into two steps: the generation step and the attack step. In this way, the transfer attack means that the targets specified in the two stages are not the same. Tables 5 and 6 show attack methods in MNIST and CIFAR-10. In order to test the transferred and non-transferred settings of the existing method, three batches of samples were obtained by specifying three targets in the generation step. Each batch of samples specifies three target models in the attack step. On the other hand, unlike existing methods, since our AWTM does not specify any targets in the generation step, there is no concept of transfer attacks. Therefore, AWTM only generates a batch of samples, then use them to specify the three target models in the generation step.

**Table 5:** Success rate on MNIST.

Attack on	FNN			LeNet			ResNet18		
Generated on	FNN (%)	LeNet (%)	ResNet18	FNN (%)	LeNet (%)	ResNet18 (%)	FNN (%)	LeNet (%)	ResNet18 (%)
FGSM	**99.38**	66.47	19.45	29.57	**99.72**	8.21	1.59	6.76	**44.30**
JSMA	**99.12**	30.21	20.04	28.37	**95.74**	6.31	13.15	18.63	24.34
DEEPFOOL	**96.78**	69.00	17.94	40.28	**98.53**	4.60	22.51	19.59	11.79
PGD	**97.31**	75.24	21.71	66.58	**89.84**	12.86	4.14	7.44	**51.36**
CW	**98.19**	40.50	21.87	9.74	**99.20**	2.27	3.15	4.32	**54.64**
advGAN	**98.21**	55.28	54.71	43.33	**98.58**	70.58	8.85	16.72	**61.06**
AWTM	81.78%			77.77%			38.49%		

**Note:**

Data in bold indicates the success rate of attacks that exceeding AWTM.

**Table 6:** Success rate on CIFAR-10.

Attack on	CNN			ResNet9			ResNet34		
Generated on	CNN (%)	ResNet9 (%)	ResNet34 (%)	CNN (%)	ResNet9 (%)	ResNet34 (%)	CNN (%)	ResNet9 (%)	ResNet34 (%)
FGSM	**99.99**	**89.36**	**88.43**	**89.47**	**100**	**90.30**	**85.98**	**89.18**	**99.98**
JSMA	**95.48**	54.92	68.93	54.06	**75.93**	66.17	73.41	69.75	**85.84**
DEEPFOOL	**100**	85.77	85.54	**82.91**	**100**	**85.78**	63.03	69.61	**99.99**
PGD	**91.77**	81.94	81.89	**80.22**	**92.54**	**81.09**	57.15	**95.89**	**85.41**
CW	**100**	80.37	82.06	**83.62**	**100**	**84.53**	53.29	52.42	**100**
advGAN	**90.52**	69.26	83.92	72.33	**91.15**	**87.12**	63.71	64.77	**93.03**
AWTM	87.99%			75.15%			76.35%		

**Note:**

Data in bold indicates the success rate of attacks that exceeding AWTM.

Under the non-transfer setting, most of the methods involved in the comparison have achieved the highest success rate. And under non-transfer settings, they are stronger than AWTM. However, non-transfer settings need to query the target, while AWTM does not. Therefore, a fairer comparison should be made between AWTM and transfer settings of other methods. Tables 5 and 6 show that AWTM is stronger than transfer settings of most other methods.

**Figure 8:**
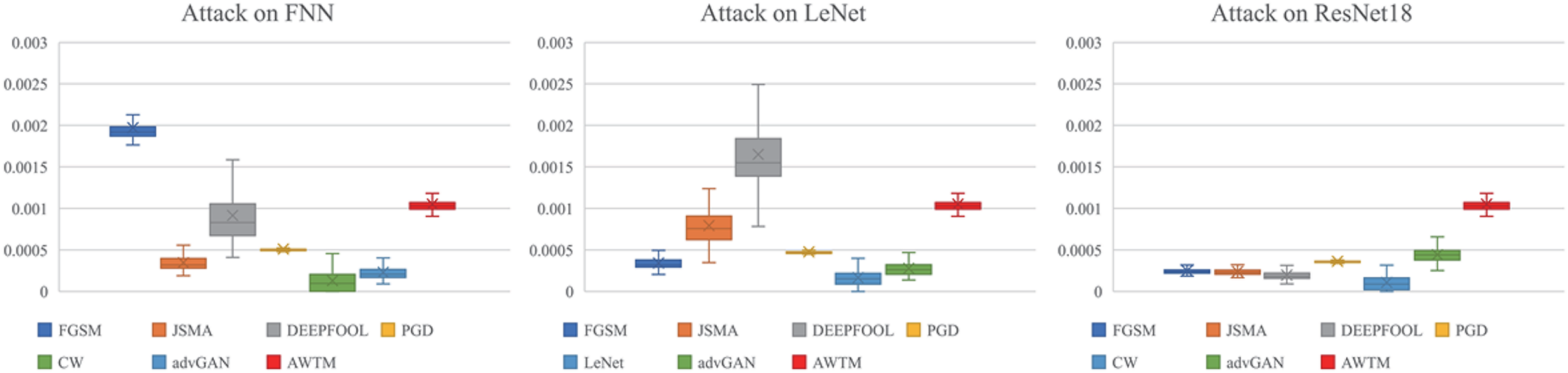
The distribution of the mean square error between the adversarial examples and the originalsample on MNIST.

**Figure 9:**
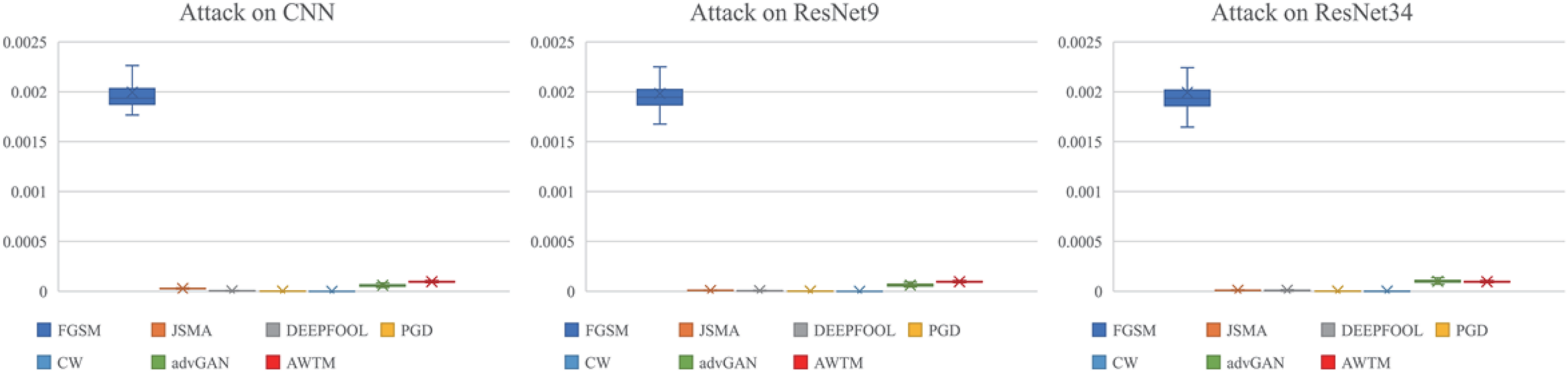
The distribution of the mean square error between the adversarial examples and the originalsample on CIFAR-10.

While testing the attack success rate, we also evaluated the quality of the adversarial examples. Figs. 8 and 9 show the distribution of the distance between the samples generated by different methods and the original samples under the mean square error measurement. Although the sample quality generated by AWTM is ordinary, it is relatively stable. When a large number of adversarial examples are generated, the AWTM ensures that their quality gap will not be too large.

In addition, we tested the attack success rate of these samples on the target models under the protection of adversarial training. As shown in Tables 7 and 8, for FNN, the benefits of adversarial training are not obvious. Both AWTM and a variety of existing methods A high attack success rate was achieved. Compared with FNN, the effect of adversarial training on LeNet and ResNet18 is very good due to their more complex network structure. However, the two adversarial training methods have less impact on AWTM on the MNIST dataset than the existing methods. In Tables 9 and 10, on the more complex CIFAR-10 data set, the attack success rate of JSMA and advGAN on the protected model exceeds our AWTM. This means that the ability of AWTM to process complex data sets needs to be improved.

**Table 7:** Success rate on MNIST (the target protected by FGSM adversarial training).

Attack on	FNN			LeNet			ResNet18		
Generated on	FNN (%)	LeNet (%)	ResNet18 (%)	FNN (%)	LeNet (%)	ResNet18 (%)	FNN (%)	LeNet (%)	ResNet18 (%)
FGSM	3.77	61.06	12.61	3.78	3.38	5.28	0.84	1.15	0.54
JSMA	44.60	26.97	12.35	11.79	17.51	5.25	10.55	8.99	3.22
DEEPFOOL	33.47	76.36	10.25	20.79	7.88	3.03	20.69	11.35	0.70
PGD	2.21	72.32	14.93	8.50	5.35	7.05	2.00	2.94	0.84
CW	6.02	21.15	7.85	4.80	4.96	6.16	3.87	1.59	0.46
advGAN	61.92	68.06	61.50	6.81	6.96	15.56	1.29	2.02	2.48
AWTM	88.56%			31.43%			37.30%		

**Table 8:** Success rate on MNIST (the target protected by PGD adversarial training).

Attack on	FNN			LeNet			ResNet18		
Generated on	FNN (%)	LeNet (%)	ResNet18 (%)	FNN (%)	LeNet (%)	ResNet18 (%)	FNN (%)	LeNet (%)	ResNet18 (%)
FGSM	29.36	79.50	9.28	6.38	23.29	4.91	1.99	3.96	3.25
JSMA	33.49	24.69	6.71	14.36	25.03	3.81	6.97	10.63	4.67
DEEPFOOL	43.45	73.18	4.64	26.31	37.90	1.73	23.12	19.31	0.84
PGD	59.67	**84.43**	18.73	23.75	**38.85**	7.90	5.77	7.57	3.56
CW	10.85	12.74	2.33	4.64	7.01	1.89	1.40	3.98	4.96
advGAN	56.04	82.21	76.03	12.71	**39.72**	**58.15**	4.83	11.08	**48.89**
AWTM	82.33%			37.81%			32.01%		

**Note:**

Data in bold indicates the success rate of attacks that exceeding AWTM.

**Table 9:** Success rate on CIFAR-10 (the target protected by FGSM adversarial training).

Attack on	CNN			ResNet9			ResNet34		
Generated on	CNN (%)	ResNet9 (%)	ResNet34 (%)	CNN (%)	ResNet9 (%)	ResNet34 (%)	CNN (%)	ResNet9 (%)	ResNet34 (%)
FGSM	20.51	33.58	32.30	44.51	3.77	46.97	24.80	26.84	14.97
JSMA	**81.47**	**80.44**	**57.16**	**81.89**	**80.41**	**59.06**	**80.62**	**80.01**	**54.95**
DEEPFOOL	25.38	29.15	28.30	30.14	17.64	32.07	21.15	22.25	19.72
PGD	24.82	26.37	26.52	27.17	18.72	27.63	20.70	20.71	19.06
CW	22.00	26.05	25.38	28.64	30.94	31.49	20.57	20.03	21.85
advGAN	34.31	34.95	**44.19**	55.25	**65.00**	**72.22**	29.05	30.07	34.08
AWTM	41.72%			57.92%			36.55%		

**Note:**

Data in bold indicates the success rate of attacks that exceeding AWTM.

**Table 10:** Success rate on CIFAR-10 (the target protected by PGD adversarial training).

Attack on	CNN			ResNet9			ResNet34		
Generated on	CNN (%)	ResNet9 (%)	ResNet34 (%)	CNN (%)	ResNet9 (%)	ResNet34 (%)	CNN (%)	ResNet9 (%)	ResNet34 (%)
FGSM	30.19	36.05	33.36	45.93	13.15	47.51	31.93	34.60	31.90
JSMA	**80.77**	**80.01**	**55.70**	**81.37**	**79.83**	57.31	**80.95**	**79.46**	**55.27**
DEEPFOOL	24.57	26.84	26.41	29.30	17.97	30.11	22.93	24.88	23.33
PGD	23.45	23.50	23.49	25.33	16.98	25.74	20.85	21.18	20.75
CW	21.68	18.57	19.26	18.46	19.74	15.34	20.83	18.47	21.56
advGAN	30.22	31.41	40.16	**60.32**	**63.14**	**74.13**	33.49	36.28	**49.34**
AWTM	40.87%			59.21%			43.39%		

**Note:**

Data in bold indicates the success rate of attacks that exceeding AWTM.

Overall, AWTM still has certain advantages. The parameters of the model after adversarial training are completely different from the previous ones. Therefore, the success rate of adversarial examples over-fitting to the previous parameters is greatly reduced. However, the classification boundaries of models trained on the same dataset will not differ greatly, and the AWTM learns the average classification boundary, so it can have a high attack success rate on these protected models.

Finally, on a machine equipped with an NVIDIA RTX2060 graphics card, we compare the generation time cost of each method, as shown in Table 11. The trained AWTM has an extremely fast generation speed, and its time cost is mainly concentrated in the training phase of the threat model. Since the training cost will not increase with the generation scale, the speed advantage of AWTM will be more salient when processing a large number of samples. For more information on all experiments, refer to https://github.com/moxiaoyugithub/AWTM.

**Table 11:** Comparison of the average time cost of each method.

	FGSM	JSMA	DEEPFOOL	PGD	AWTM
Cost on MNIST (s)	197.37	3,479.93	1,347.22	294.08	23.95
Cost on CIFAR-10 (s)	342.99	2,771.07	1,131.12	1,069.66	100.97

## Conclusion

In order to generate adversarial examples without querying the target, we proposed AWTM. It is a non-targeted, grey-box adversarial attack method, and the attacker only needs to know the target dataset to complete the generation. We generated adversarial examples and compared them with several typical generation methods on the MNIST and CIFAR-10 datasets. The results show that our AWTM is as powerful as the common methods that need to specify and query the target. However, the performance of AWTM does not seem to be good enough on complex data sets. We hope that the more advanced GAN structure in AWTM can improve its generation ability to achieve complex tasks for this problem. On the other hand, there may be a better strategy to simulate changing classification boundaries, which will greatly affect the attack’s success rate. These problems will be improved in future research.

## Appendix a

## Additional Information and Declarations

### Data Availability

The following information was supplied regarding data availability: The scripts are available at GitHub https://github.com/moxiaoyugithub/AWTM. The MNIST dataset is available at http://yann.lecun.com/exdb/mnist/. The CIFAR-10 dataset is available at http://www.cs.toronto.edu/~kriz/cifar.html.
